# Bendable solid-state supercapacitors with Au nanoparticle-embedded graphene hydrogel films

**DOI:** 10.1038/srep40163

**Published:** 2017-01-11

**Authors:** Kyungwhan Yang, Kyoungah Cho, Dae Sung Yoon, Sangsig Kim

**Affiliations:** 1 Korea University, Department of Electrical Engineering, Seoul 02841, Korea; 2 Korea University, School of Biomedical Engineering, Seoul 02841, Korea

## Abstract

In this study, we fabricate bendable solid-state supercapacitors with Au nanoparticle (NP)-embedded graphene hydrogel (GH) electrodes and investigate the influence of the Au NP embedment on the internal resistance and capacitive performance. Embedding the Au NPs into the GH electrodes results in a decrease of the internal resistance from 35 to 21 Ω, and a threefold reduction of the IR drop at a current density of 5 A/g when compared with GH electrodes without Au NPs. The Au NP-embedded GH supercapacitors (NP-GH SCs) exhibit excellent capacitive performances, with large specific capacitance (135 F/g) and high energy density (15.2 W·h/kg). Moreover, the NP-GH SCs exhibit comparable areal capacitance (168 mF/cm^2^) and operate under tensile/compressive bending.

In recent years, bendable electrical double layer capacitors—or supercapacitors—have been regarded as one of the most promising energy storage devices, owing to their fast charge/discharge rates and favorable cycle lifetimes when compared with batteries^1^,^2^,^3^,^4^. Unlike batteries, which store energy through electrochemical reactions in the electrode bulk, supercapacitors store energy primarily at the interface of the electrode and electrolyte. High-performance supercapacitors are therefore evaluated by whether or not electrode materials are sufficiently conductive to transport charge carriers to the electrolyte interface. To date, research on supercapacitor electrodes has been concentrated on the development of materials with high specific surface areas, rather than on materials with sufficient electrical conductivity^5^,^6^,^7^. Graphene hydrogel (GH) has recently been in the spotlight as a promising electrode material for supercapacitors, owing to its 3D porous network, which leads to a high specific surface area^8^,^9^,^10^. However, it has a downside in that the agglomeration of graphene sheets in GH results in a decrease of electrical conductivity and an increase of internal resistance. The graphene agglomeration is an undesirable but inevitable by-product obtained during electrode fabrication. The internal resistance comprehends the electrolyte resistance, the resistance of the electrode active-material, and the contact resistance between the active materials and the current collector^11^,^12^, and is responsible for an ohmic (IR) drop—potential drop—that has adverse effects on the supercapacitor’s power density, high-rate capability, and applicability scope. Therefore, to fabricate supercapacitors without an IR drop, it is imperative to enhance the electrical conductivity of the GH electrodes and to reduce the contact resistance between the electrodes and the current collectors^13^. Therefore, in this study, we attempt to reduce the internal resistance by embedding Au nanoparticles (NPs) into the GH electrodes. Furthermore, we examine the characteristics of the resulting supercapacitors under tensile and compressive stresses, to confirm the possibility of their use as bendable energy storage devices.

## Methods

**Figure 1:**
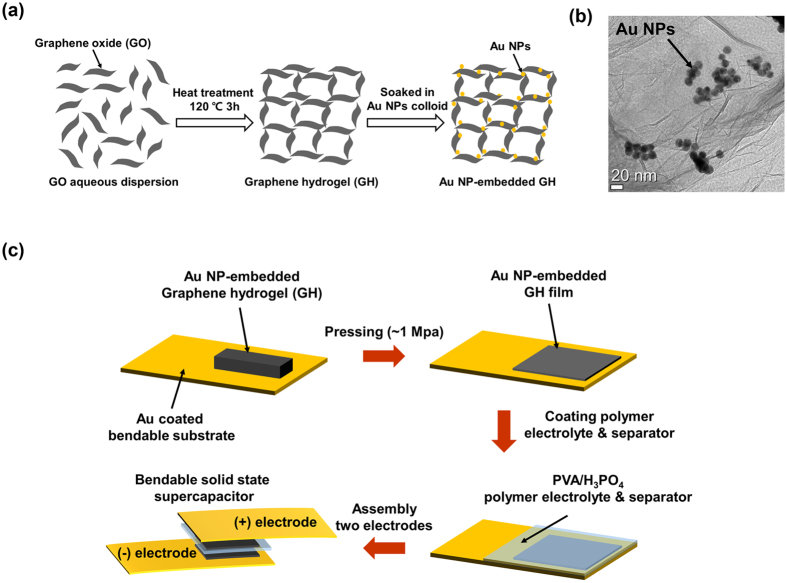
(**a**) Schematic illustration of the Au NP-embedded GH structure. (**b**) HR-TEM image of Au NP-embedded GH. (**c**) Fabrication process of the bendable solid-state NP-GH SC.

Figure 1(a) shows a simple process to obtain Au NP-embedded GH electrodes. Graphene oxide (GO) dispersed in H_2_O with a concentration of 2 mg/mL, L-glutathione powder, and a colloid with 15 nm diameter Au NPs were purchased from Sigma-Aldrich. L-glutathione, used as a reducing agent, was dissolved in a GO aqueous dispersion under stirring for 20 min, at 90 °C. After heating the mixed dispersion at 120 °C for 3 h without stirring and freeze-drying it for 12 h, a GH cylinder was produced. The obtained GH was soaked in a colloid of Au NPs with an optimized volume ratio of 1:1 to GO aqueous dispersion for 1 h and air-dried for 1 h, thereby producing the Au NP-embedded GH (supplemental information). After the preparation of the Au NP-embedded GH electrode, we investigated the existence of Au NPs in the compressed graphene sheets as well as the surface and cross-sectional morphologies by a high-resolution scanning electron microscopy (HR-SEM) (supplementary information). Figure 1(b) shows a high-resolution transmission electron microscopy (HR-TEM) image of Au NPs embedded in an interlayer of graphene sheets. The Au current collectors, with a thickness of 100 nm, were formed by thermal deposition on bendable polyethylene terephtalate (PET) substrates. A gel electrolyte was prepared using H_3_PO_4_ and polyvinyl acetate (PVA). One gram of H_3_PO_4_ was added to 10 mL of deionized water, and 1 g of PVA powder was then added to the solution. The mixture was stirred at 90 °C until the solution became clear. To fabricate the bendable electrodes, the prepared NP-embedded GH was cut into strips with a dried weight of 0.5 mg including Au NPs and pressed on the Au-coated PET substrates under a pressure of ~1 MPa, as shown in Fig. 1(c).

Subsequently, the NP-embedded GH electrodes were dipped into the (1 M) H_3_PO_4_-PVA aqueous solution, and air-dried at 50 °C for 2 h, to evaporate the excess water. The two Au NP-embedded GH electrodes were then pressed together under a pressure of ~0.3 MPa for 30 min. To investigate the effect of embedding Au NPs in the GH electrode, we prepared a supercapacitor using GH without Au NPs, to be used as a reference sample. Hereafter, the supercapacitors made of GH with and without Au NPs are referred to as NP-GH and GH supercapacitors (SCs), respectively. A GH SC was fabricated with the same processes used for the NP-GH SCs, except for the NP embedding step. The physical properties of GH and NP-GH composites were examined by x-ray diffraction (XRD) spectroscopy (Bruker, D8 Focus), Raman spectroscopy (Horiba Jobin Yvon, LabRam ARAMIS IR2) and x-ray photoelectron spectroscopy (XPS; AXIS ULTRADLD spectrophotometer). The Raman spectra were taken by the laser wavelength of 532 nm with a power of 0.5 mW. The morphologies of the NP-GH composites were characterized by field-emission scanning electron microscopy (Hitachi, S-4300) and HR-TEM (Tecnai F20). Cyclic voltammetry (CV), galvanostatic charge/discharge, and electrochemical impedance tests of the supercapacitors were carried out with an IviumStat electrochemical workstation. In addition, the mechanical stability of the NP-GH SCs was examined with homemade tensile/compressive stages with a curvature radius of 18 mm. Furthermore, three NP-GH SCs were assembled in series to confirm that the connected NP-GH SCs were able to turn on a red light-emitting diode (LED) with a minimal operating turn-on potential of 2.7 V.

## Result and Discussion

**Figure 2:**
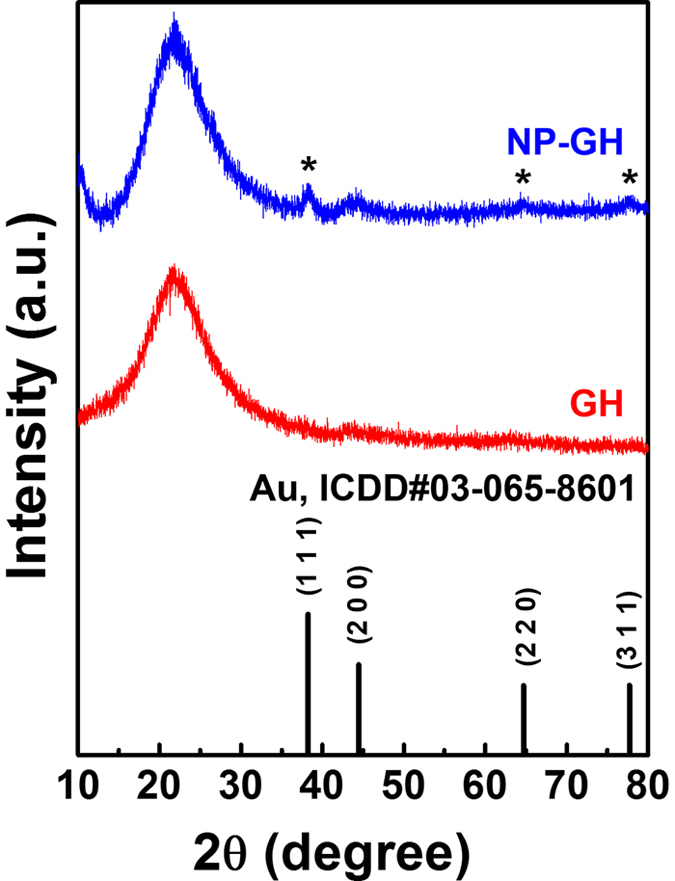
XRD patterns of the GH and the Au NP-embedded GH. The 2θ values of an Au reference (ICCD#03-065-8601) are marked in this figure.

The XRD patterns of the GH and the Au NP-embedded GH are plotted in Fig. 2 as a function of 2θ; the 2θ values of an Au reference (ICDD #03-065-8601) are marked in this figure. The peaks at 22° in these XRD patterns are identical to that of GH^14^. Compared with the pattern of the GH, some additional peaks are present at 38.4°, 64.2°, and 77.8° in the pattern of the Au NPs-embedded GH. These 2θ values are identical to those of the Au ICDD, indicating the existence of Au NPs in the GH.

**Figure 3:**
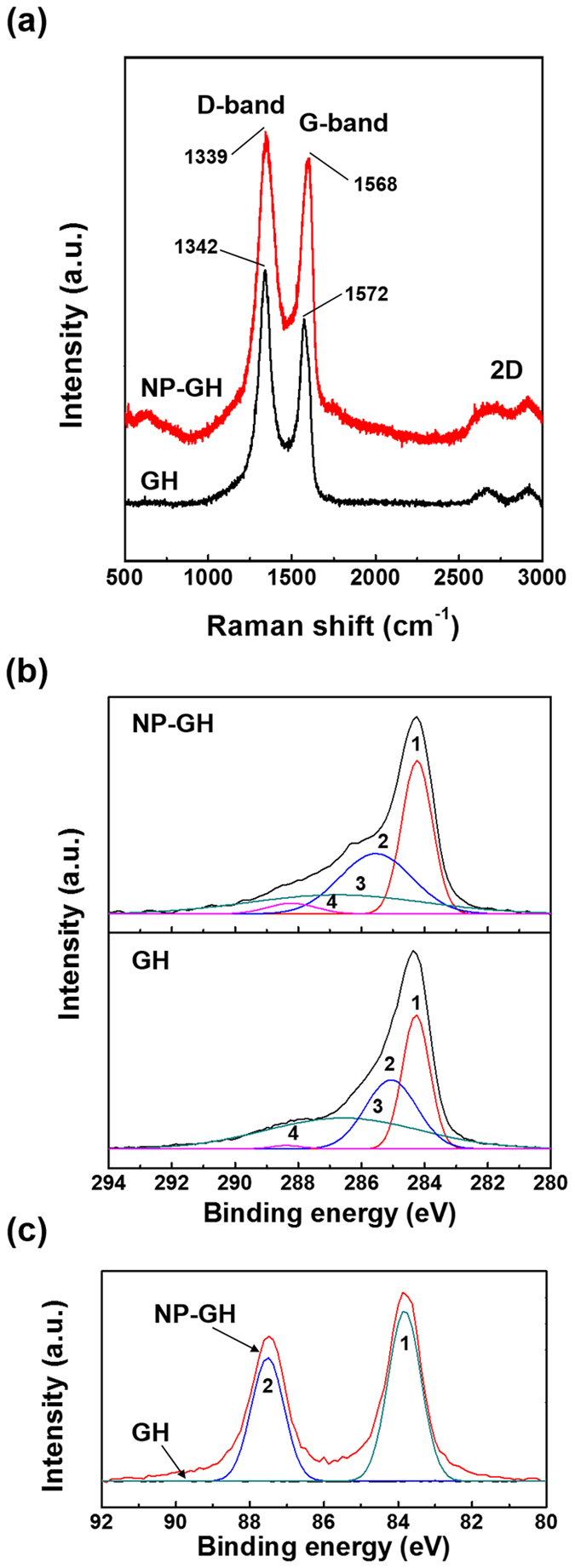
(**a**) Raman spectra of the GH and the NP-GH. XPS spectra of (**b**) C 1 s and (**c**) Au 4 f for the GH and the NP-GH.

The Au NPs in the NP-GH are interacted with carbon-carbon bonds in the GH as represented in Raman and XPS spectra in Fig. 3. The comparison of Raman spectra of the GH and the NP-GH in Fig. 3(a) reveals that the D, G, and 2D bands of the NP-GH are slightly red-shifted in Raman band position from those of the GH. This observation is consistent with the previous study on graphene impacted by Au NPs^15^. The local strain in the GH accounts for the red shift, which originates from the charges transfer between the Au NPs and the GH. And the C1s XPS spectra (Fig. 3(b)) show the increase in the concentration of sp^2^ carbon (peak 1) from 30.5 to 36.2% which is the evidence of the charge transfer. This increase in the sp^2^ carbon concentration can be estimated from the fitting of the C 1 s spectra with four different peak curves associated with C=C, C−C, C−O/C−O−C, and C=O bonds: C=C (284 eV, sp^2^; peak curve 1), C−C (284.8 eV, sp^3^; peak curve 2), C−O/C−O−C (286 eV, hydroxyl and epoxy groups; peak curve 3) and C=O (289 eV, carbonyl group; peak curve 4)^16^. And the Au 4 f spectra (Fig. 3(c)) were fitted with the widely reported peaks of 4f_5/2_ (84 eV, peak curve 1) and 4f_7/2_ (87 eV, peak curve 2)^17^. There is no peak related to Au NPs in the spectrum of the GH, which is consistent with the XRD result.

**Figure 4:**
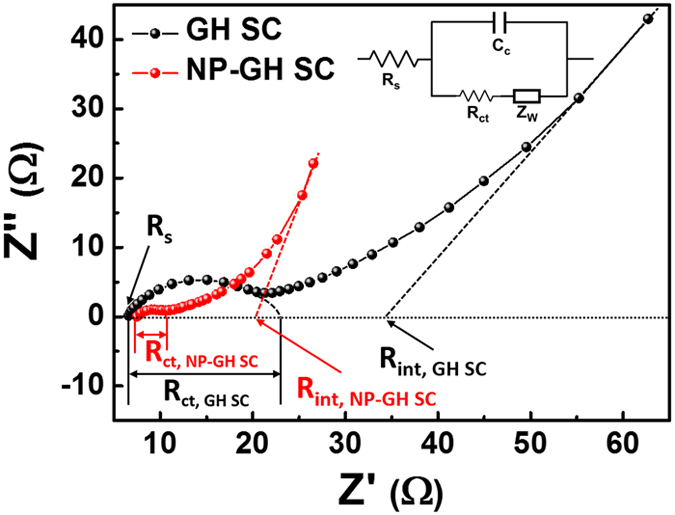
Nyquist plots of the GH SC and NP-GH SC.

Figure 4 shows the Nyquist plots of a GH SC and an NP-GH SC in the 0.1 Hz to 1 MHz frequency range. The inset shows the Randle’s equivalent electrical circuit, which consists of a series resistance (R_S_), an interfacial contact capacitance (C_C_; related to a charge accumulation at the electrode/electrolyte interface), a charge-transfer resistance (R_ct_), and a Warburg impedance (Z_W_; representing the diffusion impedance). The left part (lower Z′ region) and right part (higher Z′ region) of the plot correspond to the higher and lower frequency regions, respectively. The intersection of the represented semicircle with the Z′ axis indicates R_S_ and is mainly related to the bulk electrolyte. Both the GH SC and the NP-GH SC are constructed with the same electrolyte and separator, and therefore there is no real distinction between their R_S_ values. The diameter of the semicircle in the higher frequency region of the Nyquist plot is linked to R_ct_, including both the electronic and ionic resistances. The electronic resistance depends on the electrical conductivity of the active electrode material (the GH or NP-GH under study) and the electrical contact at the interface of the electrode material with the Au current collector. The ionic resistance is determined by the ionic conductivity in the electrode-electrolyte interface. On the other hand, the x-intercept in the lower frequency region of the Nyquist plot indicates the internal resistance (R_int_) of the SC^18^. Compared with the GH SC (R_ct_ = 16 Ω and R_int_ = 35 Ω), the NP-GH SC has relatively lower values of R_ct_ (3 Ω) and R_int_ (21 Ω), owing to the embedding of the high conductivity Au NPs. In particular, the relatively lower R_int_ of the NP-GH SC is due to the increase in the number of conducting paths by the addition of Au NPs; the additional conducting paths between an active electrode material and the current collector lower the internal resistance^19^,^20^.

**Figure 5:**
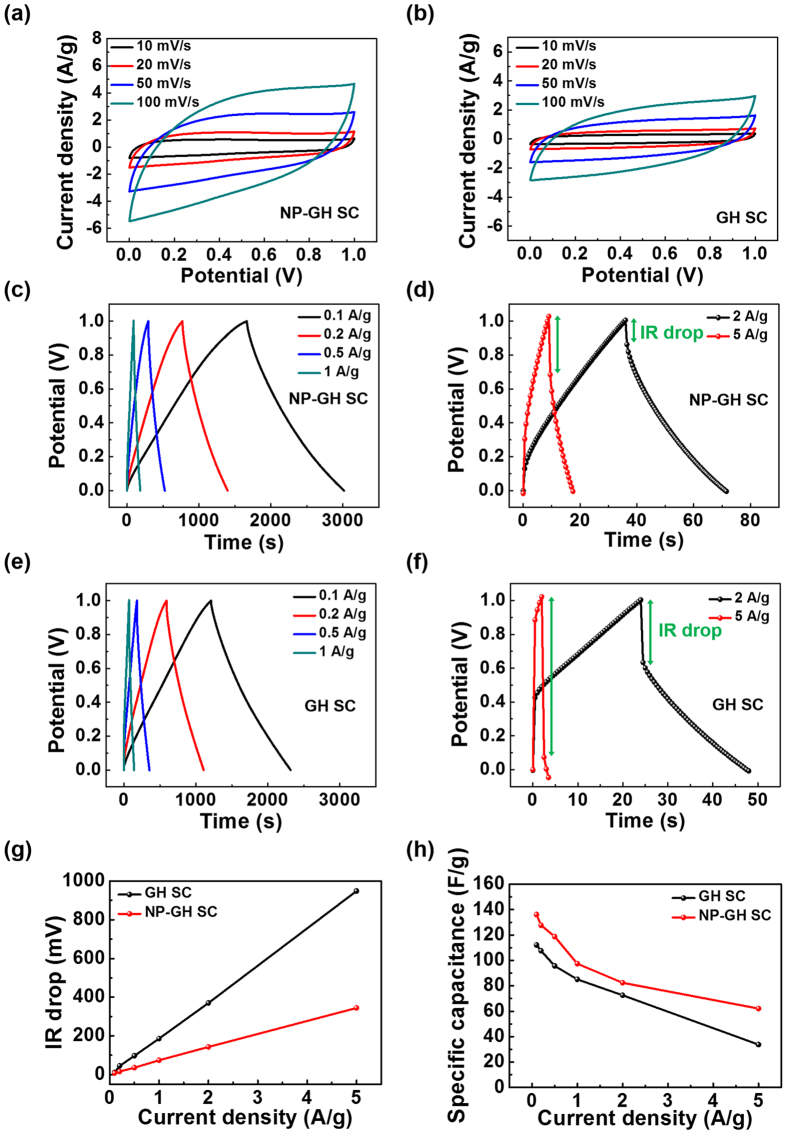
Cyclic voltammetry curves of (**a**) NP-GH SC and (**b**) GH SC. (**c–f**) Galvanostatic charge/discharge curves of (**c,d**) NP-GH SC and (**e,f**) GH SC. (**g**) IR drop as a function of current density. (**h**) Specific capacitance of both the NP-GH SC and GH SC for various current densities.

The CV of both the NP-GH SC and the GH SC were measured in the range of 0 to 1.0 V at various scan rates, as shown in Fig. 5(a) and (b), respectively. The areas enclosed by the CV curves of the NP-GH SC are larger than those of the GH SC for all scan rates, indicating that the NP-GH SC has superior performance. The high conductivity of the Au NP-embedded GH electrodes plays a role in the increase of the charge transfer speed at the electrodes^21^. Consequently, the NP-GH SC shows a sharper current response for all scan rates, indicating an improved electroactivity. The electroactivity is governed by the speed of insertion-desertion of charges through the polymer matrix, and the number of active sites offered by the electrode. Hence, the NP-GH SC has higher electroactivity than the GH SC. The same phenomenon is observed in the galvanostatic charge/discharge (GCD) curves. Figure 5(c) and (d) show the GCD curves for the NP-GH SC, and Fig. 5(e) and (f) show the GCD curves for the GH SC; these curves are presented for various current densities. At a low current density of 0.1 A/g, the GCD curves of both SCs show nearly symmetric triangular shapes. As the current density increases, the IR drop in the GCD curve for the GH SC becomes larger than that of the NP-GH SC. The IR drops of both SCs are plotted in Fig. 5(g) as functions of the current density. When the current density reaches 5 A/g, the IR drop of the GH SC is 948 mV, nearly three times larger than that of the NP-GH SC. The lower IR drop of the NP-GH SC indicates that the NP-GH SC has a relatively lower internal resistance, which agrees with the results of the Nyquist plots shown in Fig. 4. Figure 5(h) shows the specific capacitances of both NP-GH SC and GH SC, which are obtained from the equation C = I∙Δt/(m∙ΔV), I being the applied current, Δt the discharging time, m the total mass of each electrode, and ΔV the discharge potential excluding the IR drop. The energy density is calculated from the equation E = 1/2∙C∙ΔV^2^. At a current density of 0.1 A/g, the specific capacitance and energy density of the NP-GH SC are 135 F/g and 18.5 W·h/kg, respectively, which are close to the values reported in previous studies^22^,^23^. As the current density increases to 5 A/g, the specific capacitance and energy density of the NP-GH SC decrease to 62 F/g and 3.7 W·h/kg, respectively. On the other hand, the specific capacitance and energy density of the GH SC are 110.5 F/g and 15.2 W·h/kg at a current density of 0.1 A/g, but they decrease dramatically to 38 F/g and 0.014 W·h/kg as the current density increases to 5 A/g.

**Figure 6:**
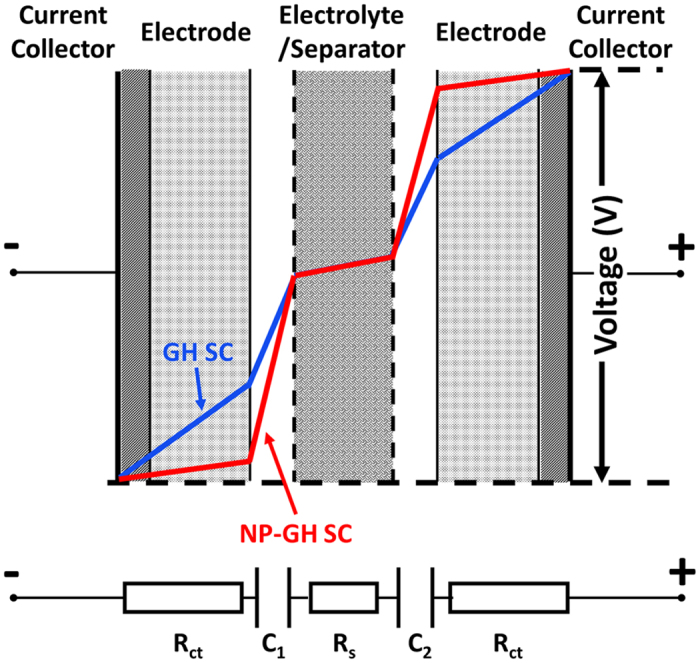
Conceptual model of the voltage distribution profile of an SC in the fully charged state.

Based on the above-described results, we propose a conceptual model for the voltage distribution profile of an SC in the fully charged state; the model can be seen in Fig. 6, where C_1_ and C_2_ designate the double layer electrical capacitances, and R_ct_ and R_s_ indicate the charge transfer resistance and series resistance, respectively, as per Fig. 4. An instantaneous voltage drop, known as IR drop, occurs when the SC switches from charging to discharging, as a result of the combined ohmic resistance of the electrodes, electrolyte, and contact resistances in the system. The total usable voltage window (ΔV) is therefore reduced by the IR drop. Both the contact resistance between the current collector and the electrode and the resistance of the electrode itself are relatively lower in the NP-GH SC, because of the conductive Au NPs. As a consequence, the ΔV of the NP-GH SC is larger than that of the GH SC, as can be seen on the voltage distribution profiles.

**Figure 7:**
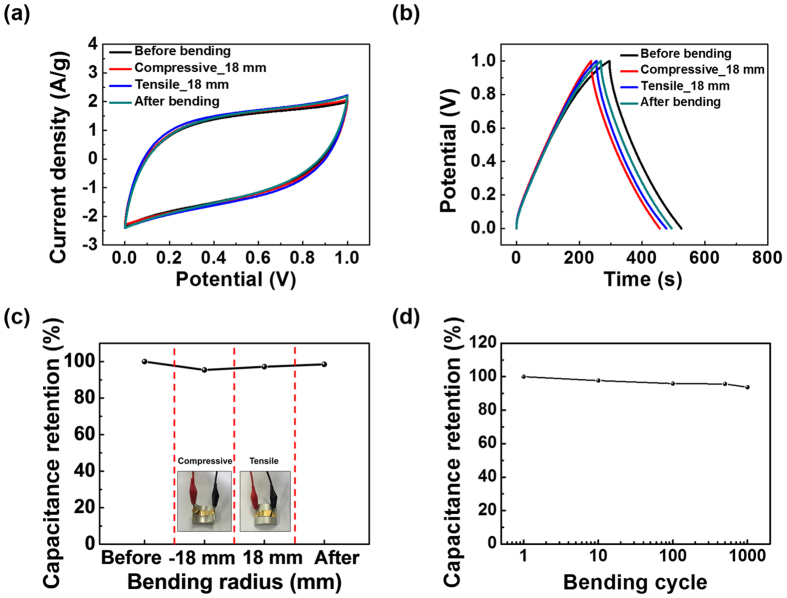
Bending stability of the NP-GH SC under tensile/compressive stress with a curvature radius of 18 mm. (**a**) Cyclic voltammetry curves. (**b**) Galvanostatic charge/discharge curves. (**c**) Normalized capacitance under various bending states. (**d**) Capacitance retention ratio as a function of the bending cycle.

Figure 7 exhibits the characteristics of the NP-GH SC as a function of the bending state. The CV curves at a scan rate of 20 mV/s show nearly the same capacitive behavior regardless of the bending state, as shown in Fig. 7(a). While the GCD curves at a current density of 0.5 A/g show small differences in the charging/discharging times (see Fig. 7(b)), the NP-GH SC has good capacitance stability with more than 98% retention after tensile/compressive bending, as shown in Fig. 7(c). The insets of Fig. 7(c) are photographs of the NP-GH SC on the tensile/compressive bending stages. Figure 7(d) represents the capacitance retention ratio, obtained from the galvanostatic charge/discharge curves and Nyquist plot of NP-GH SCs (supplemental information), as a function of the bending cycle. The capacitance retention ratio of 94% still remains even after 1000 bending cycles.

**Figure 8:**
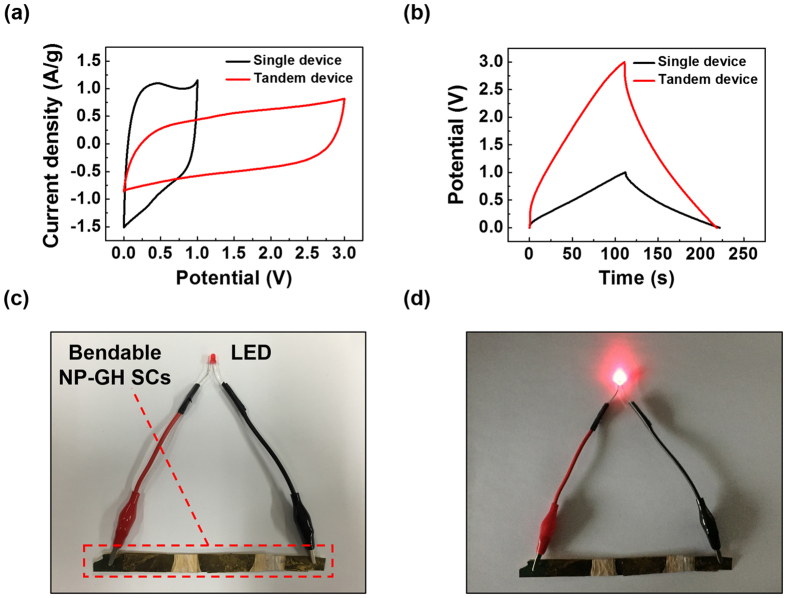
(**a**) Cyclic voltammetry curves at 20 mV/s and (**b**) galvanostatic charge/discharge curves at 0.5 A/g for both a single SC and three NP-GH SCs connected in series. (**c,d**) Photographs of a red LED connected to the tandem device (**c**) before and (**d**) after turn on.

To demonstrate the possibility of practical usage of the NP-GH SCs, we investigated the electrochemical performances of a tandem SC composed of three NP-GH SCs. As shown in Fig. 8(a) and (b), the potential window of 1.0 V for a single device was extended to 3.0 V for the tandem SC, indicating that the individual NP-GH SCs maintained their capacitive performance in the tandem configuration. Figure 8(c) and (d) show that the fully charged tandem SC was capable of turning on a red light-emitting diode (LED) with a working voltage of 2.7 V. This demonstrates the great potential of solid-state NP-GH SCs as energy storage devices in practical applications.

## Conclusions

In this study, we fabricated bendable solid-state SCs with Au NP-embedded GH electrodes, and compared their performance with a GH SC (without any embedded Au NPs). The NP-GH SC showed excellent capacitive performances with a smaller IR drop, larger specific capacitance, higher rate capability, and higher energy density. This is attributed to the reduction of the contact resistances between the electrode and the current collector and of the electrode itself, which results from the embedment of Au NPs into the GH electrodes. Furthermore, the bendable NP-GH SC operates stably under tensile/compressive bending.

## Additional Information

**How to cite this article**: Yang, K. *et al*. Bendable solid-state supercapacitors with Au nanoparticle-embedded graphene hydrogel films. *Sci. Rep.* **7**, 40163; doi: 10.1038/srep40163 (2017).

**Publisher's note:** Springer Nature remains neutral with regard to jurisdictional claims in published maps and institutional affiliations.

## Supplementary Material

Supplementary Information
