# Supplementary material for: Bendable solid-state supercapacitors with Au nanoparticle-embedded graphene hydrogel films
Source: Sci Rep. 2017 Jan 11;7:40163. doi: 10.1038/srep40163 (PMC5225469; doi:10.1038/srep40163)
Supplement: Supplementary Information [file srep40163-s1.pdf]

Supplemental information for

Bendable solid-state supercapacitors with Au nanoparticle-embedded graphene hydrogel films

Kyungwhan Yang, Kyoungah Cho, Sangsig Kim
Department of Electrical Engineering, Korea University, 146, Anam-ro, Sungbuk-gu, Seoul 02841, Korea

Dae Sung Yoon
School of Biomedical Engineering, Korea University, 146, Anam-ro, Sungbuk-gu, Seoul 02841, Korea

1. Morphologies of the Au NP-embedded GH electrode

Figure S1 represents the morphology of the NP-embedded GH electrode ((a)~(c)) and its energy dispersive spectroscopy (EDS)(d). The FE-SEM and images reveal that the Au NPs with an average dimeter of 15 nm are randomly distributed in the compressed graphene sheets. Our EDS analysis identified the materials embedded in the graphene to be Au.

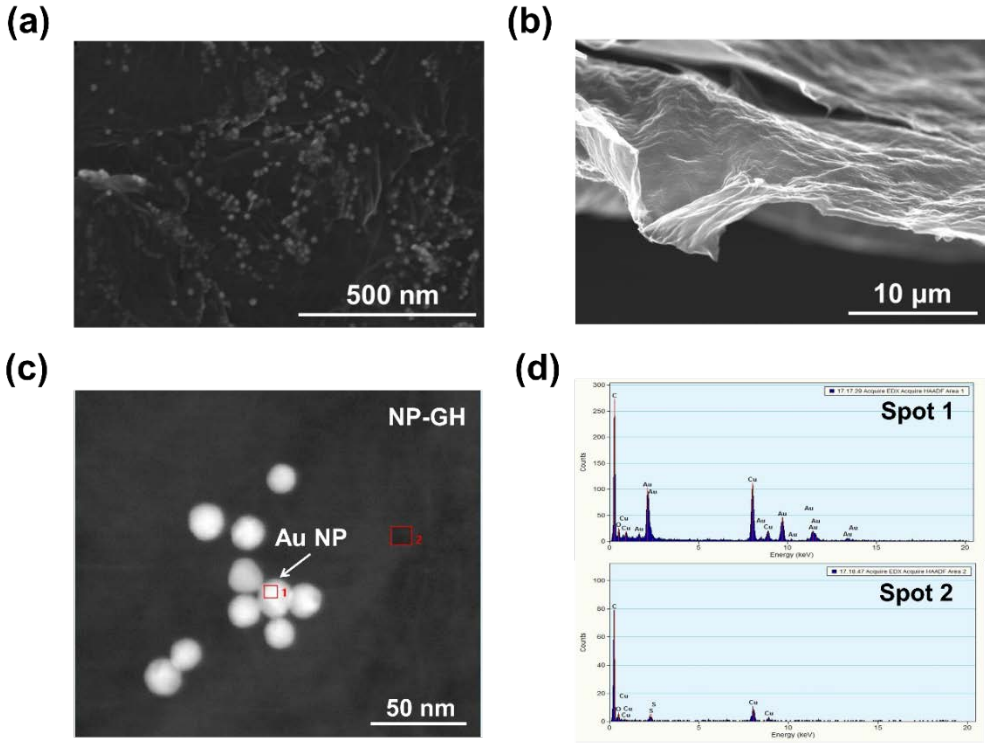

Figure S1. (a) Top surface and (b) cross-sectional FE-SEM images of the NP-GH electrode. (c) HR-SEM image of the NP-GH surface and (d) EDS spectra of Au NPs (spot 1) and graphene (spot 2).

2. Optimization of the loading level of Au NPs in NP-GH electrode

The loading level of the Au NPs is one of significant parameters affecting on the internal resistance of the electrodes and the properties of our supercapacitors. Hence, we carried out some additional experiments about the capacitive performance of the NP-GH SCs with various loading level of Au NPs. The NP-GH had been produced with Au NP-colloid of 20 mL in our previous manuscript so that the NP-GHs were produced with Au NP-colloid of 10 and 30 mL in the additional control experiments. The volume ratios of Au NP-colloid to GH were 50, 100, and 150%, respectively, for Au NP-colloid of 10, 20, and 30 mL. Figure S2 exhibits the capacitive performances of the NP-GH SCs as a function of the volume ratio of Au NP-colloid to GH. As the volume ratio increases, the IR drop decreases from 47 to 11 mV, while the specific capacitance shows the maximum value of 162 F/g when the volume ratio of Au NP-colloid to GH is 100%. This observation implies that the overdose of Au NPs on the GH electrode brings about the reduction of the effective charging area of GH and consequently the capacitive performances are degraded. Hence, the optimized volume ratio of the Au NP-colloid to GH is 100% in this study.

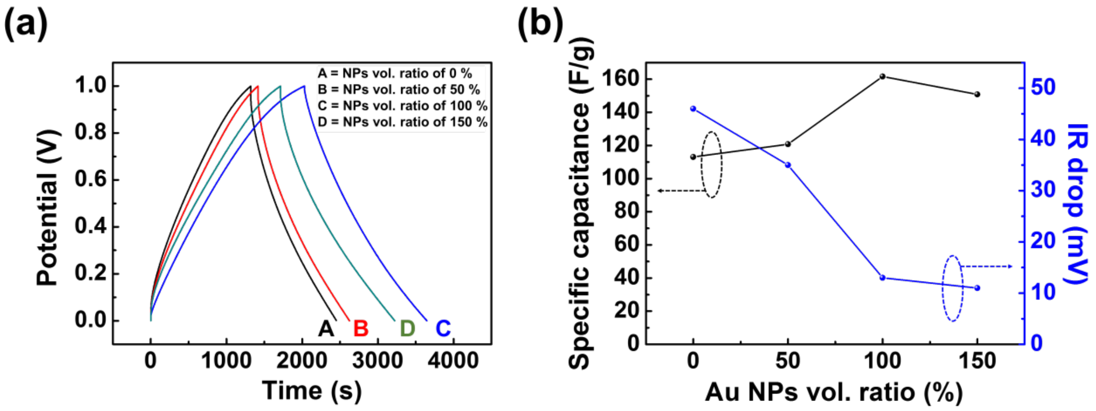

Figure S2. (a) Galvanostatic charge/discharge curves of NP-GH SCs with different volume ratios of Au NP-colloid. (b) Specific capacitance and IR drop plots of NP-GH SCs with different volume ratios of Au NP-colloid.

3. Bending cycle performances of the NP-GH SCs

We examined the cycle performance of the NP-GH SC under tensile stress with a curvature radius of 18 mm. Figure S3 shows the characteristics of the NP-GH SC as a function of the bending cycle, revealing that the capacitance retention ratio of 94% still remains even after 1000 bending cycles.

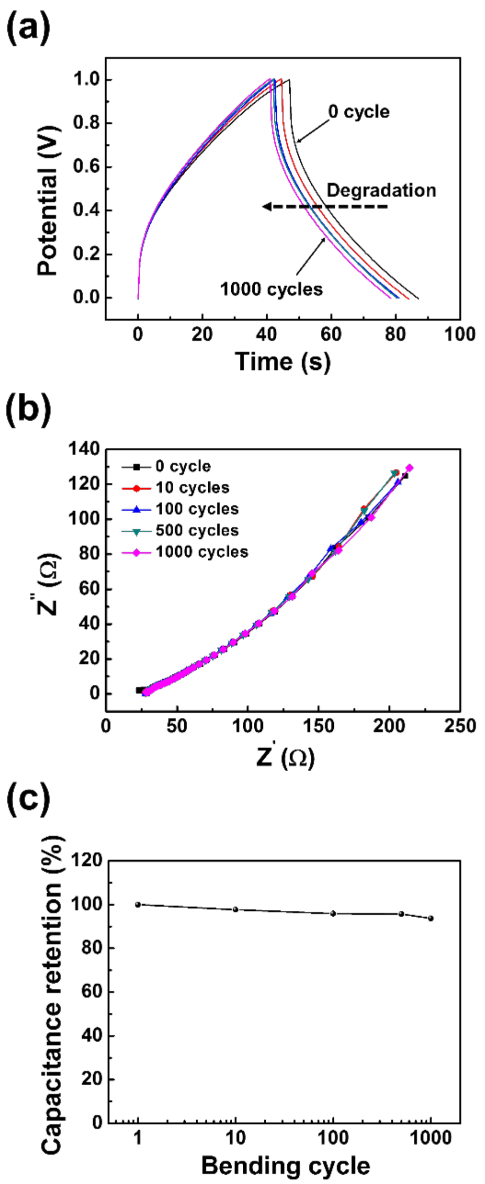

Figure S3. (a) Galvanostatic charge/discharge curves, (b) Nyquist plots and (c) Capacitance retention ratio as a function of the bending cycle.
